# Co-Designing a Smoking Cessation Chatbot: Focus Group Study of End Users and Smoking Cessation Professionals

**DOI:** 10.2196/56505

**Published:** 2024-08-19

**Authors:** Hollie Bendotti, Sheleigh Lawler, David Ireland, Coral Gartner, Henry M Marshall

**Affiliations:** 1 Thoracic Research Centre Faculty of Medicine The University of Queensland Brisbane Australia; 2 Australia e-Health Research Centre Commonwealth Scientific and Industrial Research Organisation Brisbane Australia; 3 School of Public Health Faculty of Medicine The University of Queensland Brisbane Australia; 4 National Health and Medical Research Council Centre of Research Excellence on Achieving the Tobacco Endgame School of Public Health The University of Queensland Brisbane Australia; 5 Department of Thoracic Medicine The Prince Charles Hospital Metro North Hospital and Health Service Brisbane Australia

**Keywords:** artificial intelligence, chatbot, smoking cessation, behavior change, smoking, mobile health, apps, digital interventions, smartphone, mobile phone

## Abstract

**Background:**

Our prototype smoking cessation chatbot, Quin, provides evidence-based, personalized support delivered via a smartphone app to help people quit smoking. We developed Quin using a multiphase program of co-design research, part of which included focus group evaluation of Quin among stakeholders prior to clinical testing.

**Objective:**

This study aimed to gather and compare feedback on the user experience of the Quin prototype from end users and smoking cessation professionals (SCPs) via a beta testing process to inform ongoing chatbot iterations and refinements.

**Methods:**

Following active and passive recruitment, we conducted web-based focus groups with SCPs and end users from Queensland, Australia. Participants tested the app for 1-2 weeks prior to focus group discussion and could also log conversation feedback within the app. Focus groups of SCPs were completed first to review the breadth and accuracy of information, and feedback was prioritized and implemented as major updates using Agile processes prior to end user focus groups. We categorized logged in-app feedback using content analysis and thematically analyzed focus group transcripts.

**Results:**

In total, 6 focus groups were completed between August 2022 and June 2023; 3 for SCPs (n=9 participants) and 3 for end users (n=7 participants). Four SCPs had previously smoked, and most end users currently smoked cigarettes (n=5), and 2 had quit smoking. The mean duration of focus groups was 58 (SD 10.9; range 46-74) minutes. We identified four major themes from focus group feedback: (1) conversation design, (2) functionality, (3) relationality and anthropomorphism, and (4) role as a smoking cessation support tool. In response to SCPs’ feedback, we made two major updates to Quin between cohorts: (1) improvements to conversation flow and (2) addition of the “Moments of Crisis” conversation tree. Participant feedback also informed 17 recommendations for future smoking cessation chatbot developments.

**Conclusions:**

Feedback from end users and SCPs highlighted the importance of chatbot functionality, as this underpinned Quin’s conversation design and relationality. The ready accessibility of accurate cessation information and impartial support that Quin provided was recognized as a key benefit for end users, the latter of which contributed to a feeling of accountability to the chatbot. Findings will inform the ongoing development of a mature prototype for clinical testing.

## Introduction

In 2019, a total of 1.1 billion people worldwide smoked tobacco regularly, and 13.6% of all deaths were attributable to smoking tobacco [1]. Tobacco’s burden on human health is compounded by the chronic relapsing-remitting nature of tobacco dependence [2]; 96%-97% of people are unsuccessful when trying to quit without assistance [3]. Smoking cessation behavioral support increases individual quit attempt success [4] and increases further when pharmacotherapy is used, supporting the physiological and behavioral needs of the individual [5]. However, the scalability and reach of professional interventions such as behavioral counseling are limited due to accessibility [6,7] and individual awareness [8,9]. Digital interventions, such as smartphone apps, have therefore been explored to improve the reach of smoking cessation information and behavioral support. However, there is currently limited evidence to support the routine use of smartphone apps in smoking cessation [10-12], and questions remain surrounding the impact of the level of engagement with an app on its effectiveness.

The use of conversational agents for smoking cessation is an emerging area of research that may, through personalization and tailoring, enhance engagement with digital smoking cessation interventions, thereby increasing their impact. Conversational artificial intelligence (AI), such as chatbots, allows for synchronous communication with users via text and/or audio using natural language processing and machine learning algorithms with a rule-based and/or probabilistic approach [13]. Over time with increasing use and data generation, these interactions may become more natural, responsive, and tailored to the user [13]. Mohr’s model of “Supportive Accountability” states that engagement with digital health interventions is promoted with the addition of human support by cultivating a sense of personal accountability to a competent, trustworthy, and caring coach [14]. Chatbots capable of emulating this type of human support via a highly accessible platform such as a smartphone app may bridge the gap between scalable personalized interventions and effective behavioral counseling. Furthermore, the current evidence supporting the effectiveness of conversational AI interventions on smoking cessation outcomes is limited but promising [15-17], and previous studies have found that they are generally an acceptable tool among participants [13,18-20].

**Figure 1:**
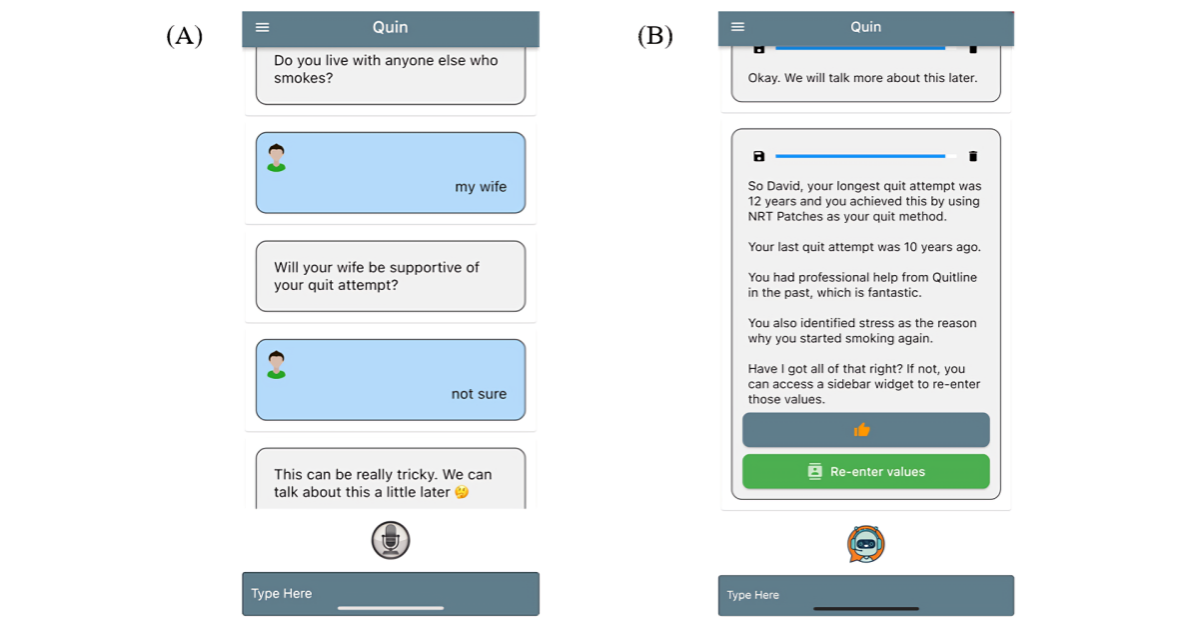
(A) Quin user interface; (B) Example of personalization in Quin dialogue.

Quin is a prototype chatbot that aims to improve access to personalized, evidence-based smoking cessation information and support via an app. Quin has been developed as part of a multiphase program of co-design research by a multidisciplinary team. Quin was designed using a “bottom-up approach,” in that we analyzed and applied findings from real-world evidence-based counseling interactions (ie, consumer- and stakeholder-driven research) rather than a top-down approach by applying behavior change theories or frameworks from the outset. A detailed description of the design and development of the Quin prototype has been published elsewhere [21] and a screenshot of Quin’s user interface is provided as Figure 1. Our co-design approach is based on a 3-stage model developed by the Good Things Foundation (National Health Service, UK Government), which aims to promote digital inclusion [22]:

- Stage 1: Define user and stakeholder needs and experiences
- Stage 2: Ideas and prototype
- Stage 3: Iterative testing and delivery

The design foundations of Quin are embedded in stage 1 by understanding and translating user and stakeholder needs and experiences at 2 levels, the platform or user interface (ie, smartphone app) and the content (ie, smoking cessation counseling and education). To understand the user experience of existing mobile smoking cessation (mCessation) apps, we first analyzed unsolicited user reviews of apps to determine important design recommendations across domains of app personalization, relationality, functionality, and credibility [23]. To understand the counselor-patient relationship and interaction, we analyzed real-world Quitline counseling sessions to identify conversation themes and topics, including how topics map to conversation stages and specific statements, questions, and responses from counselors and clients [21, 24]. Quitline counselors are trained to deliver cognitive behavioral therapy strategies and/or motivational interviewing alongside evidence-based smoking cessation information during counseling conversations.

We applied these findings to Quin’s conversation design and technical development (stage 2). The types of human-computer interactions with Quin include conversing (via text or speech-to-text), instructing (eg, user selecting options), and responding (eg, notifications or initiating the conversation upon opening) [25]. Quin’s dialogue is structured into “initial” and “support” conversations. The initial conversation constructs a personalized “quit plan” from demographic, smoking, and quit histories (Figure 1) to guide a personalized discussion about pharmacotherapy options and behavioral considerations or support. Based on the agreed quit plan, follow-up conversations are scheduled to check in on users’ progress and review and to answer questions and troubleshoot issues [21]. Quin was programmed using a collection of natural language processing algorithms within CSIBot, the Commonwealth Scientific and Industrial Research Organisation health chatbot framework. The primary response system is a case-based reasoner, which applies both syntactic matching and sentiment analysis algorithms [21]. Examples of these algorithms are presented in Figure 1. The syntactic matching algorithm uses a radix tree data structure to store and search for a template response that can include wildcard substitutions and extra chatbot operations (eg, log data) [21]. As such, the conversation is guided and tailored by free-text and preset responses which Quin can store and recall to determine subsequent conversation paths.

At all times, we aimed to keep the language and responses very positive and affirming. For example, if the user states that the cost of smoking is a motivator for quitting smoking, Quin reinforces this by saying “Extra cash is a great reward for quitting smoking! Are you saving up for something?” Where applicable within the conversation, Quin also provides links to relevant external resources such as educational videos and Quitline services and generates a “to-do” list based on the user-defined quit plan. For example, if the user has indicated they are interested in using a prescription medication, the list will include “Speak to your doctor about using [medication].” Users also have the option to upload motivational images during the initial conversation, and Quin uses information collected within the conversation to inform a cost analysis to track savings and editable summary of user profile information.

Having developed the prototype Quin (stage 2), we now present the first phase of iterative testing (stage 3), which sought to understand how stakeholders perceive and interact with Quin, a fundamental step for ongoing phases of iterative development and refinement prior to clinical efficacy trials. Our data collection included qualitative inquiry to provide a deeper understanding of the user experience, end user preferences, and priorities, as this is an important aspect of human-centered design. As such, this study aimed to gather feedback on the Quin prototype from end users and smoking cessation professionals (SCPs) via a beta testing process to (1) identify factors that positively and negatively influence the user experience, including general views on chatbot technology for smoking cessation and suggestions for improvement; and (2) compare and contrast subjective feedback from end users and SCPs. Acknowledging the importance of applied consumer-driven research, we will then seek to apply the findings to produce a mature prototype for clinical testing.

## Methods

This focus group study was completed in Queensland, Australia, and collected qualitative data from focus groups, and feedback logged within the chatbot app.

### Ethical Considerations

Ethics approval was granted by The Prince Charles Hospital Human Research Ethics Committee (HREC Project ID: 69623). Written informed consent was obtained from all participants before taking part in the study, and they were free to withdraw at any time. End user participants who completed a focus group were given an Aus $40 (US $26.65) gift card. Focus group transcripts were deidentified prior to analysis, and logged in-app feedback was anonymous.

### Recruitment and Participants

**Figure 2:**
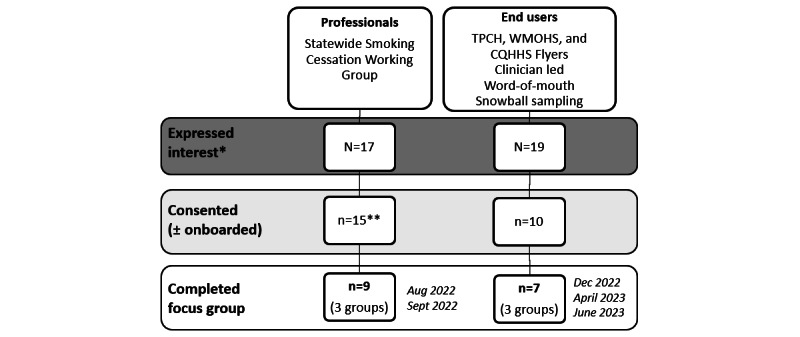
Focus group recruitment flow diagram. CQHHS: Central Queensland Hospital and Health Service; TPCH: The Prince Charles Hospital; WMOHS: West Moreton Hospital and Health Service. *Contacted the study team (phone or email) and further information and consent form provided, **9 professionals onboarded to the Quin app.

Multiple methods of active and passive recruitment were used. SCPs from the Queensland State Health Department (Statewide Smoking Cessation Working Group and Quitline) were invited to participate via email distribution lists. End user recruitment was broad and included priority populations for smoking cessation assistance, such as people from regional or rural areas, older people with respiratory conditions or diseases, and people from low-income areas. Targeted recruitment included electronic flyer distribution via a regional public health unit to associated community organizations and physical flyer distribution via clinicians and visual display within respiratory clinics and mental health units at The Prince Charles Hospital and dental clinics at West Moreton Hospital and Health Service. We also recruited end users via word of mouth and snowballing methods. Inclusion criteria for end user participants were people aged 18 years and older who self-identify as currently smoking, either not ready or attempting to quit smoking, or have recently quit smoking (within the previous 12 months); were English-speaking; and owned a smartphone (Apple or Android) and could download apps. Participants who expressed interest were sent an electronic participant information and consent form via the survey platform REDCap (Research Electronic Data Capture; Vanderbilt University) hosted at The University of Queensland. Following informed consent, participants were sent an electronic survey to collect demographic and smoking and quit history information. The recruitment process is outlined in Figure 2.

### Beta Testing Procedures and Focus Groups

Consenting participants were onboarded to the Quin app 1 to 2 weeks prior to their scheduled focus group. Onboarding meetings with the lead researcher (HB) or a research assistant were completed via videoconferencing with a summary email of written instructions or only via a summary email if a participant was not available to videoconference. These meetings allowed the research team to assist with downloading the beta version and to provide general testing instructions to maximize the collection of user feedback. Users were encouraged to create multiple demographic profiles to test chatbot responses to different combinations of information and were instructed how to log feedback within the app if anomalies were found (eg, spelling errors and incorrect responses). Logged feedback was stored on a Google Firebase server. Study team members were available to assist with any issues during the testing period.

SCP focus groups were completed first to ensure the accuracy and breadth of smoking cessation information provided within Quin. Following prioritization and incorporation of feedback from SCPs as major updates, end user focus groups were completed. All focus groups were held via videoconferencing. To improve anonymity, participants had the option to leave their camera off and use their first name only (eg, username and in discussion). The focus groups were moderated by the lead investigator (HB), an early career researcher who has formal training and experience in qualitative research as well as expertise in health promotion and public health. Senior researchers SL and/or HMM were present during focus groups to take notes and assist with probing or follow-up questions where applicable. SL is an experienced qualitative researcher with expertise in health psychology, health promotion, and public health. HMM is a thoracic medicine specialist with a bachelor of psychology (honors) and research interests in smoking cessation interventions. At the end of the focus group, upon departure of participants, the investigators remained in the meeting to reflect on the session, feedback, and notes taken. The focus group sessions followed a Human Research Ethics Committee–approved interview guide (Multimedia Appendix 1) based on the categories of feedback within the end user version of the Mobile App Rating Scale [26] but allowed for probing and follow-up questions to expand on discussion. End users were asked exploratory questions regarding smoking and quitting histories and barriers and enablers to smoking cessation as well as experiences, if any, using mCessation apps; SCPs were asked similar questions reframed as their experience of what clients commonly tell them. The moderator ensured all participants were given an opportunity to speak and expand on others’ ideas. Focus groups were recorded, transcribed verbatim, and deidentified prior to analysis. All participants had the option to continue testing the app and provide additional feedback after their focus group.

### Analysis

Quantitative data from demographic surveys were analyzed descriptively. In-app feedback was downloaded from Google Firebase after completion of all SCP focus groups and after each end -user focus group. This feedback was categorized using content analysis, reviewed, and prioritized for incorporation into Quin by all investigators.

Focus group transcripts were imported into NVivo software (version 12; Lumivero), and analysis was completed in 2 ways. First, after completion of all SCP focus groups, transcripts and notes were reviewed by HB to identify key improvements to be made prior to end user focus groups. These findings were presented, discussed, and agreed upon by our multidisciplinary research team before updates to Quin were made. This approach is reflective of Agile development methods, and we present major changes to Quin using theme, epic, stories, and tasks [27]. Second, following the completion of all focus groups, transcripts were analyzed inductively by HB and SL using thematic analysis [28]. To address the aim of the study, 1 researcher (HB) broadly coded data into categories of positive and negative feedback and suggestions for improvement. Both study team members then generated an initial set of codes within each category, which were reviewed and combined to define and refine themes and subthemes. Disagreements in data interpretation and theme development were able to be resolved via discussion between HB and SL, but a third researcher (HMM) was available to discuss differences in interpretation during thematic analysis. We present the themes and subthemes, including direct quotes, by aforementioned categories and compare and contrast feedback between professionals and end users.

## Results

### Overview

Three focus groups of 9 SCPs were completed in August (n=4) and September (n=3; n=2) 2022 testing Quin (beta version 21). Three focus groups of 7 end users were completed in December 2022 (n=3) and April (n=2) and June (n=2) 2023, testing Quin beta versions 28, 30, and 31, respectively. The mean duration of focus groups was 58 (SD 10.9; range 46-74) minutes. Participant demographic characteristics are summarized in Table 1. Occupations of SCPs included clinical nurses, occupational therapists, pharmacists, Quitline telephone counselors, and health promotion officers across metropolitan, rural, and Indigenous health services, of which the majority (n=8) delivered smoking cessation counseling and advice regarding nicotine replacement therapy. Most end users currently smoked cigarettes on a daily (n=3) or less than daily basis (n=2), and 2 had quit smoking. Nicotine dependence scores were calculated using the Fagerström Test for Nicotine Dependence [29], but results may be unreliable, as most end users anecdotally indicated they had fully or partially (ie, dual use) transitioned to e-cigarettes (either as a tobacco product or cessation aid) and answered the questions in the context of cigarette smoking.

**Table 1:** Participant demographics by cohort.

Item		Professionals (n=9)	End users (n=7)
**Age (years), n (%)**			
	25-29	—^a^	2 (29)
	30-39	—	2 (29)
	40-49	7 (78)	1 (14)
	50-59	1 (11)	1 (14)
	60-69	1 (11)	—
	70+	—	1 (14)
**Sex, n (%)**			
	Female	9 (100)	2 (29)
	Male	—	5 (71)
**Postcode, n (%)**			
	Major city	7 (78)	5 (71)
	Inner regional	1 (11)	1 (14)
	Remote	—	1 (14)
	Very remote	1 (11)	—
**Indigenous, n (%)**			
	Aboriginal	1 (11)	—
	No	8 (89)	7 (100)
**Education, n (%)**			
	Senior high school	—	2 (29)
	Trade certificate	—	2 (29)
	Bachelor degree	6 (67)	1 (14)
	Master degree	3 (33)	2 (29)
**Phone type, n (%)**			
	Apple	8 (89)	4 (57)
	Android	1 (11)	3 (43)
**Currently smoke, n (%)**			
	Daily	—	3 (43)
	Less than daily	—	2 (29)
	Not at all	9 (100)	2 (29)
**Previously smoked, n (%)**			
	Daily	3 (33)	6 (86)
	Less than daily	1 (11)	—
	Both daily and less than daily	—	1 (14)
	Never smoked	5 (56)	—
**Type of tobacco (current)^b^, n (%)**			
	Manufactured cigarettes	—	4 (80)
	Roll your own	—	2 (40)
	Pipe	—	1 (20)
	Heated	—	1 (20)
	e-Cigarettes (nonprescription)	—	1 (20)
**Type of tobacco (previously smoked)^c^, n (%)**			
	Manufactured cigarettes	2 (50)	1 (50)
	Roll-your-own cigarettes	3 (75)	2 (100)
	Cigars or cigarillos	—	1 (50)
Age start smoking (years), mean (SD)^d^		16 (4.3)	16 (4.1)
**Time to first cigarette^b^, n (%)**			
	Within 5 minutes	—	1 (20)
	6 to 30 minutes	—	2 (40)
	31 to 60 minutes	—	2 (40)
	After 60 minutes	—	—
**FTND^e,f^, n (%)**			
	Very low	—	2 (50)
	Low	—	2 (50)

^a^Not available.

^b^End users: n=5.

^c^Professionals: n=4 and end users: n=2.

^d^Professionals: n=4 and end users: n=7.

^e^Missing data: n=1 cigarette per day.

^f^FTND: Fagerström Test for Nicotine Dependence.

### Logged Feedback

In total, 46 instances of feedback were logged within Quin by 6 SCPs. Feedback related to pattern matching errors (ie, user inputs were misinterpreted or unable to be detected by the chatbot; n=13, 28%); missing conversation trees (ie, topics that require more than 1 response; n=6, 13%), smoking cessation information (n=9, 20%), and responses (ie, information to be programmed; n=8, 17%); logic issues (ie, the decision to enter a conversation tree; n=4, 9%); dialogue content (n=2, 4%); spelling or grammatical errors (n=2, 4%); notifications (n=1, 2%); and user interface (n=1, 2%). In contrast, 4 end users logged 9 instances of feedback related to logic issues (n=2, 22%), pattern matching errors (n=2, 22%), missing responses (n=2, 22%), dialogue content (n=1, 11%), notifications (n=1, 11%), and user interface (n=1, 11%).

### Major and Minor Updates to Quin Between Cohorts

**Figure 3:**
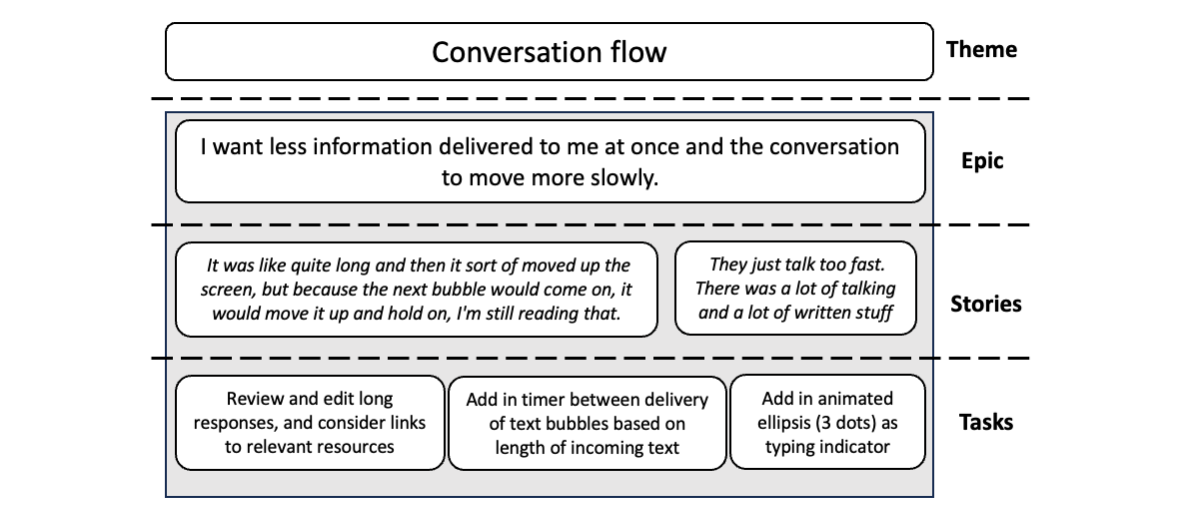
Changes to conversation flow using Agile methods.

**Figure 4:**
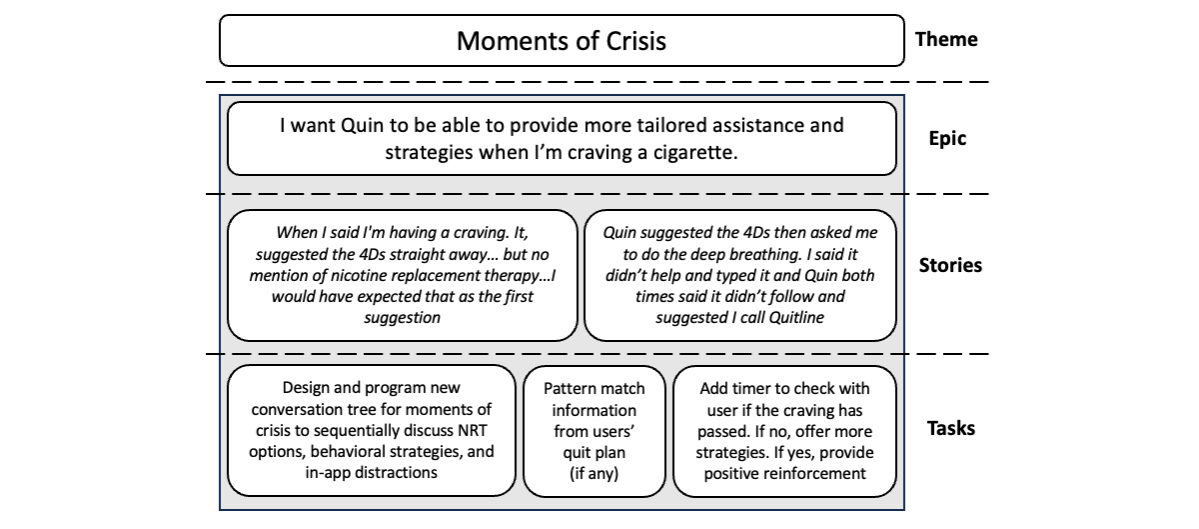
Changes to the conversation in response to acute cigarette cravings using Agile methods. NRT: nicotine replacement therapy.

Two major updates were made to the Quin prototype between versions 21 and 28 following a review of the focus group and logged feedback from the SCPs, a process illustrated in Figures 3 and 4. First, we reviewed and implemented changes to the conversation flow to improve the visual delivery of information within the app. This included consolidating information into smaller dialogue bubbles, including links to relevant resources, and slowing the delivery of information by including a timer based on the length of text and animated ellipses to indicate the chatbot “typing.” Second, we designed and implemented a conversation tree to better handle instances when a user may be craving a cigarette (ie, a “Moment of Crisis”). Quin was programmed to identify more statements that suggest an acute craving and to work sequentially through pharmacotherapy options and behavioral strategies depending on the context of the craving (ie, trigger), of which questions or responses were tailored if the user had created a quit plan. Quin was also programmed to check in with the user to see if the craving had passed and offer positive reinforcement or more options for strategies.

Other minor updates were made between versions to correct errors and improve functionality based on logged feedback, which included spelling errors, connecting programmed but inactive conversation trees, coding missing pattern matching or sentiment analysis, and updates to the chatbot dialogue and smoking cessation information.

### Feedback Themes

#### Overview

**Figure 5:**
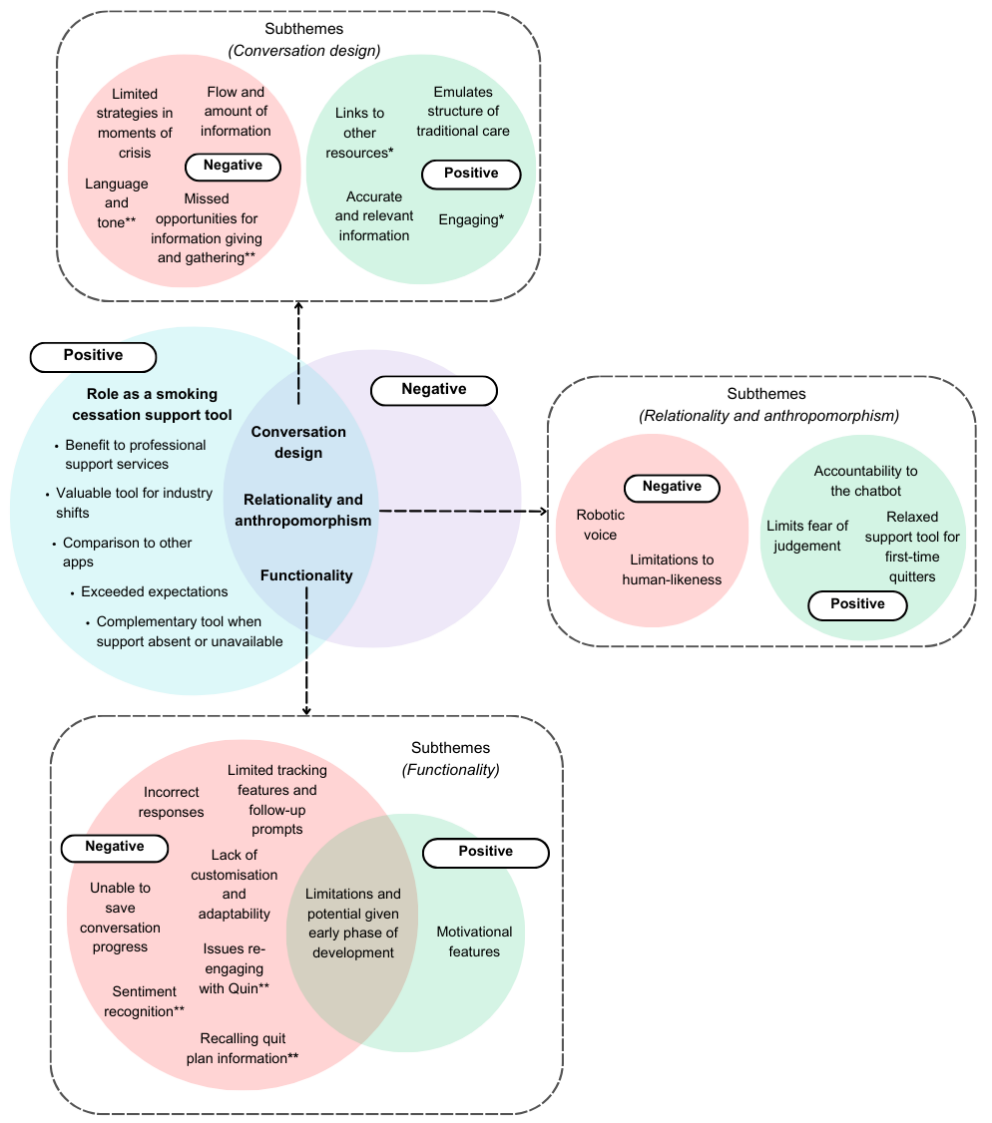
Positive and negative feedback themes and subthemes. SCP: smoking cessation professional. *End users’ feedback only, **SCPs’ feedback only.

Four high-level themes encompassing positive and negative user feedback were identified (Figure 5). While these themes are reported separately, there was a crossover between them within individuals’ feedback, given the technical connection between the features. We also provide a summary of chatbot design recommendations based on this feedback (Textbox 1).

**Summary of recommendations for smoking cessation chatbot design based on focus group feedback.**

**Smoking cessation support features**

- Provide tailored assistance in moments of acute cravings
- Ability to develop a quit plan based on previous experiences and personal preferences
- Follow-up conversations for user self-reflection on quit plan
- Proactive check-in times and notifications to enhance the sense of accountability
- Accurate educational content and cessation information
- Ability to integrate into or complement existing services (eg, preparation tool and after-hours support)
- Offer distraction and motivational tools within the conversation (eg, photo upload and tracking tools)

**Conversation design**

- Maximize opportunities for personalization and information gathering or giving
- Speed (delivery) of chatbot responses should emulate human conversation (eg, typing)
- Outsource large amounts of information by linking to other resources
- Include positive reinforcement in responses
- Limit the cyclical nature of conversation trees

**Functionality**

- Avoid errors in responses and sentiment analysis
- Include safeguards and referral pathways when the chatbot is unable to respond
- Automatically save progress within conversations
- Automatically save, store, and recall user-defined quit plan
- Ability to respond to changing preferences and information

#### Conversation Design

A key omission from the conversation reported by SCPs was Quin’s ability to provide assistance when the user is experiencing a cigarette craving by suggesting rapid-acting nicotine replacement therapy and behavioral strategies. As such, this feedback informed the inclusion of the “Moments of Crisis” conversation tree (Figure 4). The first iteration of this conversation tree was then tested by end users; however, limitations to Quin’s ability to respond correctly or appropriately in these instances were also reported.

At times, SCPs found the amount of information being delivered was too much and too fast, which disrupted the visual flow of information as they needed to scroll back through the conversation to read Quin’s responses. This feedback informed the previously described updates to the conversation flow (Figure 3). Progression through the conversation, such as when to type a response or use a radio button, was also unclear to some SCPs. After updates were made between cohorts, this issue regarding conversation flow was not raised by end users during open discussion, even when prompted.

Participants across both cohorts found the information provided within Quin to be accurate, relevant, and educational. Some end users reported a humanlike feel to the dialogue content and visual conversation flow and appreciated outsourcing large amounts of information by linking to relevant resources as a more efficient way to learn.

It did link to those videos. And one thing that I appreciated was, it was clearly YouTube content that I’d been pushed towards rather than Quin ... I’ve noticed that Quin, quite cleverly, never has more than a really easily digestible amount of prose that that comes back. And I thought that was a really cool feature.

End user 1, Focus group 5

Even the little dot dot dots to make you think that it’s typing, I think that’s really cute. Obviously it needs a moment to process, and that makes you think that you’re talking to a human, it really does feel like you’re having a chat with someone it’s not like for the most part generic robot answers ... for the most part the conversation style with the bot was really good.

End user 3, Focus group 4

End users and SCPs also thought the conversation structure emulated that of traditional care, in that users had the ability to form a quit plan alongside receiving smoking cessation information. Furthermore, reminders about aspects of their quit plan within future conversations with Quin were found to be motivating by one end user.

I really like this app because there’s the information as well as the planning side of things.

SCP 3, Focus group 2

One end user noted the chatbot conversation to be quite cyclical and scripted but also acknowledged the resources needed to expand the scope of the technology to improve this. Missed opportunities for giving and gathering information were identified by SCPs including limited prompts to see a general practitioner and asking users about motivations or reasons for previous quit attempts and caffeine and alcohol use. Finally, one SCP perceived the tone of a specific response related to quitting unassisted (ie, cold turkey) to be slightly patronizing and provided suggestions to better promote positive reinforcement. In contrast, no end users commented on the tone of the chatbot dialogue.

I found the tone of it was probably a little bit condescending. It was like, “Oh, well, you must have great willpower to do that,” and it didn’t kind of go on any more to say, “Hey, Great! That’s great. Have a try. But remember, we’ve got lots, lots of options.”

SCP 1, Focus group 2

#### Functionality

Issues with chatbot functionality recounted across cohorts were largely related to its inability to respond correctly to some user inputs, which occasionally resulted in the conversation to end abruptly. There were also instances where negative sentiment in users’ language was not recognized.

Frustration was expressed by both cohorts when progress was lost during the initial “appointment,” as the conversation did not save when they left the app and suggested to include an indication of time required to complete the first conversation. Issues re-engaging with Quin to restart the quit plan discussion and recall quit plan information were also raised.

I sort of got halfway through ... when I went back in it didn’t sort of pick up where we left off. I found myself having to start again. That was a yeah, a peeve moment for me.

End user 1, Focus group 5

End users and SCPs reported a lack of customization for user responses to some questions as well as limited adaptability of chatbot responses to changing quit preferences and information.

I’ve gone in a couple of times since then, typed in questions ... “I’m not having any luck with cold turkey. Can I follow up with something else.” And I think I got an exploding robot*, so no response. [SCP 1, Focus group 2] *Represents Quin not being able to provide a response

Despite the issues highlighted, many participants (largely end users) acknowledged the functionality and potential of Quin relative to its early phase of development and limited resources. Participants from both cohorts reported being able to complete an initial conversation and set up a quit plan. Motivational features prompted by the chatbot, such as the ability to upload images and the cost analysis of money saved per year, were valued by end users.

The app is really good in that I can pick up the app, which I have been using a lot, I look at my motivations, so what a picture of myself and my husband ... They’re really really motivating as well.

End user 2, Focus group 5

#### Relationality and Anthropomorphism

Both cohorts described limitations to Quin’s ability to emulate humanlike characteristics and how this impacted their user experience. Most participants expressed they felt like they were talking to a robot, and this feedback was often linked to functionality issues. Some end users did report they initially felt like they were talking to a human, but errors in chatbot responses impaired this effect, causing frustration, which discouraged them from continuing to use it.

End user 2: You think you’re talking to someone. And then, when that happened, you’re like, “Oh, this is just a bot,” and it actually annoys you and makes you go well exit... I’m not talking to this thing. Yeah I had that feeling a couple of times with it.

End user 1: Yeah, the same thing. You’re talking to it ... You think you’re talking to a person, and then ... it answers to something totally irrelevant. Well, I think. “Oh it’s a machine I’m talking to.”

Focus group 4

End users highlighted the benefit of Quin being an impartial proactive support for a quit attempt. Some participants expressed a sense of accountability to the chatbot and appreciated the nonconfrontational nature of the interaction, which in turn limited the fear of judgment. As such, it was a relaxed support tool for first-time quit attempts and a source of positive reinforcement.

I think it would be useful for me ... It would keep me honest. Asking me you know “How many of you smokes have you had today? When did you have your last smoke?” Things like that. Yeah, it would keep me honest.

End user 1, Focus group 4

I totally agree with ... End user 1 who said about the chatbot feature being non confronting instead of having to talk to someone. I definitely think that would help. You know, countless people who don’t really want to talk about it face-to-face with someone.

End user 2, Focus group 4

#### Role as a Smoking Cessation Support Tool

End users from all focus groups believed the chatbot app was an appropriate and acceptable support tool for smoking cessation. For some, their experience exceeded their expectations, in that they simply enjoyed using the chatbot or they got more cessation support or educational benefit from the technology than anticipated.

It was a good tool to bounce back at myself. Which I honestly can say I didn’t expect from a bot. It was, it was much more helpful than I thought it would be, even though I’m not actually in the process of quitting smoking. I still found it quite like a quite positive experience to use.

End user 1, Focus group 6

Encouragingly, an end user found it provided them the extra support they needed during their current quit attempt. Others felt that Quin was more helpful in comparison to other smoking cessation apps due to the educational content and interactivity with the chatbot. More importantly, one end user also highlighted the benefit of the potential for rapid adaptability of this technology in response to new health challenges and cessation support as a result of industry shifts (eg, vaping prevalence and cessation support).

Ease of access to smoking cessation information and support was seen as a key benefit by both cohorts. From the perspective of SCPs, the chatbot had the potential to integrate into and complement their services as both a client preparation tool and proactive complementary tool alongside professional support including when this support is unavailable (ie, after hours).

I think that that is an awesome concept for anything after hours, you know, if you’re really really struggling at like three o’clock on a Sunday morning. You can still feel like interacting and getting immediate responses rather than just, you know, reading something that’s already there printed. I think you know it’s just a good to know that you can have an answer 24/7 essentially, I think that that’s really great.

SCP 2, Focus group 3

The idea of being able to share information from the app with Quitline could be a very beneficial thing ... they’ve got the background on the client, and it’s a really straightforward-based way to start a conversation ... So I think it’s a great way to, yeah, it kind of builds rapport. It’s strength based ... and possibly cuts down on a bit of time as well, which would be valuable for the individual client as well as the service.

SCP 3, Focus group 2

## Discussion

### Principal Findings

This focus group study, with an embedded beta testing process, aimed to explore SCP and end user feedback on the user experience of a prototype smoking cessation chatbot. We found the qualitative feedback to be similar between cohorts; yet, some feedback subthemes were also exclusive to cohorts, with end users reporting more positive feedback and SCPs reporting more critical feedback. This is likely due to major updates made to Quin between cohorts but may also reflect differing priorities and expectations placed on the chatbot by SCPs and end users, which is a trend we have observed in our previous study of smoking cessation apps [23]. Overall, initial testing feedback on the user experience from both participant cohorts shows promise for Quin as an acceptable smoking cessation support tool; however, aspects of usability require improvement. Our findings build upon and reinforce previous qualitative research on user experiences with conversational agents for smoking cessation [13,18,19,30], and results will be incorporated into future iterations of Quin to produce a mature prototype for clinical testing.

### Comparison to Prior Work

Key benefits of Quin highlighted across cohorts included the smoking cessation information being relevant and acceptable and the accessibility of the chatbot as an impartial support tool. Smoking cessation often requires a multifaceted individualized approach [31], delivered over a medium- to long-term period of months to years; expanding the avenues of behavioral support is a step toward overcoming barriers to care. Consulting with SCPs allowed us not only to ensure the accuracy of the information but also to explore the potential interoperability and integration of Quin within their professional services, with many acknowledging it as a preparation tool for both the client and counselor prior to a course of care and support tool outside of service hours. Previous research has found people disclose information to and interact with chatbots similarly to how they would a human [32], and in the context of smoking cessation, that chatbot support was superior to that of family and friends [19]. Participants in our study identified Quin as supportive, nonconfrontational, and nonjudgmental. This finding reflects previous qualitative inquiries, which found that groups of sexual and gender minority young adults [30] and veterans [18] found embodied conversational agents provided nonjudgmental and/or humanlike smoking cessation support. Future first-time clients may therefore feel more comfortable providing smoking and quit histories to Quin, from which a counselor could use this information to build rapport with the client or clients may wish to only engage with Quin due to feelings of judgment or shame commonly experienced by people who smoke.

Our findings are consistent with those reported in a similar qualitative study [19], in that the sense of accountability to the chatbot reported by end users partially supports Mohr’s model of “Supportive Accountability” [14]. The model states that engagement with digital health interventions is promoted with the addition of human support by fostering a sense of personal accountability to a legitimate, trustworthy, and caring coach [14]. This qualitative study of 14 people found that engagement with, and feelings of accountability to, a smoking cessation chatbot was attributed to the humanlike features and interaction style of the chatbot alongside users’ perceived need for support [19]. However, the legitimacy and trustworthiness [14] of Quin beta versions may have been compromised by functionality issues, which impeded the relationality of the chatbot as a coach. Therefore, improved functionality must be consistent to sustain its legitimacy as a coach.

All participants across cohorts expressed negative feedback regarding the usability and user experience of Quin among aspects of conversation design, functionality, and relationality. Given the technical connection between these features, the functionality of the AI system is at the core of these identified issues. When we consider the usability goals described by Rogers et al [25], the reported inefficiencies in Quin primarily affected the utility (eg, limited tracking features and follow-up prompts) and efficiency (eg, unable to respond correctly). A key design feature of Quin is that the conversation is largely driven by free-text responses via a rule-based system to enhance user engagement and better emulate traditional human support. Previous qualitative research has also found that this approach to smoking cessation chatbot design is preferred by end users, in that responses and questions are tailored and relative to their momentary needs and situations [19]. While Quin’s current response system allows for adaptability and greater control over the accuracy of health information being provided, it is limited, in that it requires a defined structure with comprehensive coding of all potential utterances, which may increase the chance of errors, and health information needs to be reviewed and updated on a regular basis. Yet, to rely solely on a predictive model may increase the risk of incorrect information being delivered, which in the context of health behavior change could be problematic or even counterproductive, and limit the degree of personalization in responses [33]. Moreover, rule-based systems require relatively small computational and memory resources and can operate on a mobile device greatly increasing the privacy of user data with minimal operating costs. Most predictive models of suitable performance operate as a server accessible via the internet and require human utterances to be transmitted to the server potentially exposing sensitive data that may be intercepted by a hostile actor. Furthermore, there are known ethical issues and biases associated with the collation and marking of large amounts of training data required for predictive models [34], which can further compound existing inequalities (eg, sex, race, ethnicity, and socioeconomic status). However, the high flexibility of such systems is persuasive, and we believe future technological advances may resolve some of these issues. Therefore, we suggest that a carefully balanced combination of a rule-based system with a more flexible predictive model may improve the adaptability and relationality of a conversational agent intervention as a digital coach.

Contrasting feedback between cohorts following major updates lends support to the Agile development process. Significant changes made to the delivery of information within Quin were validated through end user focus group feedback, with most finding the dialogue and visual conversation flow emulated that of a human and observing specific updates (eg, animated typing ellipses and outsourcing information to links). A similar iterative development process between participant cohorts has also been used in the training of an automated motivational interviewing–based chatbot for unmotivated people who smoke [13]. The traditional path to market for medical devices and pharmaceutical products consists of 4 sequential clinical evaluation phases to determine safety, efficacy, dosing, and effectiveness [35]. In contrast, digital health product development is dynamic and nonlinear due to the rate at which technology evolves and can be modified. Agile development processes allow for greater flexibility and ability to adapt to continuous iterative feedback. Like co-design, Agile methods also prioritize collaboration between developers and end users for effective solutions [36].

### Future Directions

Finally, there are potential opportunities for Quin’s development and future role in smoking cessation services. Quin’s dialogue is continually modifiable, and the benefit of this technology is that it can be easily updated to reflect changing evidence or advice in response to industry shifts and new health challenges and tailored to target specific population groups. The Quin prototype in its current state is the foundation, upon which we plan to iteratively build using our co-design methodology to analyze Quitline cessation support for priority groups (eg, people who are pregnant) and e-cigarette use and to ultimately work backward toward a theory-informed intervention. The accessibility of Quin will allow it to complement and support existing counseling services and primary care. For example, Quin may act as a liaison to initiate cessation support on an on-demand basis after-hours or for the time between referral and initial contact, ideally with interoperability with relevant patient management or health systems to promote continuity of care. Conversely, as previously mentioned, Quin alone as the anonymous coach may be the preferred form of cessation support for people reluctant to access services.

### Strengths and Limitations

A strength of this study was the early involvement of key stakeholders in the app development process through an open and informed dialogue. This is an important stage of co-design for digital inclusion [22] and is considered a principle of digital development [37]. Furthermore, the capacity to review and incorporate key feedback between cohort groups allowed for the validation of some updates, evidenced by changes in feedback following major updates to the conversation flow. We also included a variety of end user participants across most age groups, including older age participants who are a key demographic for smoking cessation and mobile health interventions as well as participants from regional and remote locations. However, there are several limitations to this study. First, our participation rate in focus groups was low due to attrition. While we made many efforts to engage with potential participants, some did not respond after contacting the study team to express interest in participating or after consenting or were unable to attend the focus group after being onboarded to the app. As such, our recommendations for smoking cessation chatbot design are limited in their generalizability. Second, current objective nicotine dependence scores among end users were mostly low, which may limit the generalizability of our findings, as people with higher nicotine dependence may have different experiences and needs. This should also be interpreted with caution, as most end users reported in the focus groups that they had transitioned to disposable e-cigarettes, which are known to contain high levels of nicotine, and their use is not captured in the assessment. As such, the true level of nicotine dependence was likely higher among end users. Nevertheless, all end user participants, and some SCPs, had recent previous experience with daily cigarette smoking from a young age and multiple quit attempts. Third, while we encouraged open and honest feedback, a degree of social desirability bias may have influenced subjective feedback.

### Conclusions

Feedback from end users and SCPs on the user experience of the Quin prototype highlighted the importance of functionality and its impact on the conversation flow and relationality of chatbot technology. Despite this, participants recognized the benefit of Quin to provide easily accessible information, nonjudgmental support, and positive reinforcement, including how this can complement existing cessation support services. Ongoing development will incorporate these findings to produce a mature prototype for clinical testing, and the design recommendations provided may assist future smoking cessation chatbot developments.

## Appendix

Multimedia Appendix 1 Focus group interview guides.

## Abbreviations

AI: artificial intelligence
REDCap: Research Electronic Data Capture
SCP: smoking cessation professional

## Data Availability

The data sets generated and analyzed during this study are not publicly available due to ethical considerations, as the qualitative data are of personal nature and are only available to the study team on approved servers.
