# Supplementary material for: Co-Designing a Smoking Cessation Chatbot: Focus Group Study of End Users and Smoking Cessation Professionals
Source: JMIR Hum Factors. 2024 Aug 19;11:e56505. doi: 10.2196/56505 (PMC11369547; doi:10.2196/56505)
Supplement: Multimedia Appendix 1 [file humanfactors_v11i1e56505_app1.docx]

# For consumer groups

- Welcome, names. Consent & survey
- Introduce facilitators
- Explain purpose of the session – Brief background to the project (including basic introduction to the concept of mCessation apps and AI). We have invited you here today because we would like to get your feedback and suggestions on a new smartphone app that provides personalised counselling to help you quit smoking or maintain a quit attempt.

We will start with some questions about smoking and your previous experiences when attempting to quit (if any). Thank you for taking the time to use our prototype over the last few weeks and rating its quality using the uMARS tool. Today we will ask you to provide further feedback for our prototype app, as this will serve as our discussion to gather more information about what you like and don’t like about the app, and how we can improve it.

We will be audio recording your responses, but no identifying information will be reported in the results.

**Practical aspects of the session**

The session will take approximately 1 – 1.5 hours

We need to ensure only one person speaks at a time in order to hear everyone clearly on the audio tape. Please raise your hand if you would like to say something if someone is already speaking. If at any point you feel uncomfortable or upset please let us know.

Before you leave we will give you a $40 voucher to thank you for your participation

**Questions**

Please introduce yourself, tell us whether you have quit, would like to quit or have no intention to quit smoking and (icebreaker question).

**Quitting smoking (15mins)**

1. What are some reasons you would like to or did quit smoking?
2. For those who haven’t thought about quitting, what would make you want to quit? What were your main motivations?
3. What have you tried to help you quit in the past? What did you like or dislike about that method? What types of support do you prefer?
4. What forms of information do you prefer? Where do you like to get your information from?
5. How do you think smartphone apps could help you quit smoking? Has anyone used one? If so, which one, and what did you like or dislike about it?
6. What is/was your main concern about quitting smoking?

**Chatbot prototype (60 mins)**

**Questions**

*Engagement*

1. Overall, do you think this app is appropriate for those wanting to quit smoking? What aspects, if any, did you find inappropriate, unclear or confusing?
2. What specifically about the app did/didn’t make it fun or interesting to use?
3. How did you feel interacting with the app? (Prompts: language, conversational ability of the chatbot, did it feel personable or like a counsellor?)

*Functionality*

1. How easy was the app to use? What made it easy or how could it be made easier?
2. If anyone experienced problems with the app being slow or crashing, what were you trying to do/ask when this happened?

*Aesthetics*

1. What about the visual design of the app did/didn’t you like? (prompts: Easily to read? Graphics? Layout?)

*Information*

1. Did the chatbot provide relevant answers to your questions? In what instances did you think it could have provided you more information, answered your question incorrectly or answered your question very well?
2. Was there any information you were seeking but couldn’t be provided by the chatbot?
3. What are the main topics you would expect the chatbot to cover?
4. What links to more information, support and resources would you expect to see within the app?

**Final questions**

1. Do you think this app would be helpful/beneficial when trying to quit smoking, why/why not?
2. How do you think it can be improved?

Reminder: designated smoking areas and laws.

# Focus Group Guide (for professional group)

- Welcome
- Introduce facilitators
- Explain purpose of the session – Brief background to the project (including basic introduction to the concept of mCessation apps and AI). We have invited you here today because we would like to get your expert feedback and suggestions on a new smartphone app that provides personalised counselling to users quit smoking or maintain a quit attempt.

Thank you for taking the time to use our prototype over the last few weeks and rating its quality using the uMARS tool. Today we will ask you to provide further feedback for our prototype app, as this will serve as our discussion to gather more information about what you like and don’t like about the app, and how we can improve it.

We will be recording this Zoom session, but no identifying information will be reported in the results.

**Practical aspects of the session**

The session will take approximately 1.5 hours

We need to ensure only one person speaks at a time in order to hear everyone clearly on the recording. Please raise your hand if you would like to say something if someone is already speaking.

**Questions**

Please introduce yourselves and your area of expertise, and how frequently do you see patients who smoke?

**Smoking cessation experience (15-30mins)**

1. What feedback do you get from clients regarding their quit attempts, e.g. common experiences, issues and barriers?
2. What are your views on smoking cessation apps? Do you believe they have a place in supporting smoking cessation?

**Chatbot prototype (60 mins)**

**Questions**

*Engagement*

1. Overall, do you think this app is appropriate for those wanting to quit smoking? What aspects, if any, did you find inappropriate, unclear, or confusing?
2. What specifically about the app did/did not make it fun or interesting to use?
3. How did you feel interacting with the app? (Prompts: language, conversational ability of the chatbot, did it feel personable or like a counsellor?)

*Functionality*

1. How easy was the app to use? What made it easy or how could it be made easier?
2. If anyone experienced problems with the app being slow or crashing, what were you trying to do/ask when this happened?

*Aesthetics*

1. What about the visual design of the app did/didn’t you like? (prompts: Easy to read? Graphics? Layout?)

*Information*

1. Did the chatbot provide relevant answers to your questions? In what instances did you think it could have provided you more information, answered your question incorrectly or answered your question very well?
2. Was there any information you were seeking but couldn’t be provided by the chatbot?
3. What are the main topics you would expect the chatbot to cover, but it didn’t?
4. What links to more information, support and resources would you expect to see within the app?

**Final questions**

1. Would you recommend this to a client – why/why not?
2. Do you think this app would be helpful/beneficial when trying to quit smoking, why/why not?
3. Do you think this app could be of use in clinical practice? E.g connecting you with patients and their smoking information.
4. We are hoping this app can be as generalisable as possible, but do you think this needs to be tailored to specific priority populations? (i.e. cultural appropriateness)
5. Any other ideas on how you think it can be improved?
